# Wld^S^ Reduces Paraquat-Induced Cytotoxicity via SIRT1 in Non-Neuronal Cells by Attenuating the Depletion of NAD

**DOI:** 10.1371/journal.pone.0021770

**Published:** 2011-07-05

**Authors:** Qiujing Yu, Ting Wang, Xuexia Zhou, Jingxia Wu, Xingmiao Chen, Yang Liu, Dongmei Wu, Qiwei Zhai

**Affiliations:** Key Laboratory of Nutrition and Metabolism, Institute for Nutritional Sciences, Shanghai Institutes for Biological Sciences, Graduate School of the Chinese Academy of Sciences, Chinese Academy of Sciences, Shanghai, China; Tulane University Health Sciences Center, United States of America

## Abstract

Wld^S^ is a fusion protein with NAD synthesis activity, and has been reported to protect axonal and synaptic compartments of neurons from various mechanical, genetic and chemical insults. However, whether Wld^S^ can protect non-neuronal cells against toxic chemicals is largely unknown. Here we found that Wld^S^ significantly reduced the cytotoxicity of bipyridylium herbicides paraquat and diquat in mouse embryonic fibroblasts, but had no effect on the cytotoxicity induced by chromium (VI), hydrogen peroxide, etoposide, tunicamycin or brefeldin A. Wld^S^ also slowed down the death of mice induced by intraperitoneal injection of paraquat. Further studies demonstrated that Wld^S^ markedly attenuated mitochondrial injury including disruption of mitochondrial membrane potential, structural damage and decline of ATP induced by paraquat. Disruption of the NAD synthesis activity of Wld^S^ by an H112A or F116S point mutation resulted in loss of its protective function against paraquat-induced cell death. Furthermore, Wld^S^ delayed the decrease of intracellular NAD levels induced by paraquat. Similarly, treatment with NAD or its precursor nicotinamide mononucleotide attenuated paraquat-induced cytotoxicity and decline of ATP and NAD levels. In addition, we showed that SIRT1 was required for both exogenous NAD and Wld^S^-mediated cellular protection against paraquat. These findings suggest that NAD and SIRT1 mediate the protective function of Wld^S^ against the cytotoxicity induced by paraquat, which provides new clues for the mechanisms underlying the protective function of Wld^S^ in both neuronal and non-neuronal cells, and implies that attenuation of NAD depletion may be effective to alleviate paraquat poisoning.

## Introduction

The Wallerian degeneration slow (Wld^S^) mice, a spontaneous mutant mouse strain, exhibit significant neuroprotection of axons and synapses from various neurodegenerative stimuli including mechanical, genetic or chemical injury [1]–[4]. Genetic analysis has attributed this protective activity to the expression of a fusion protein, named Wld^S^, which is composed of the N-terminal 70 amino acids of ubiquitin fusion degradation protein 2a (Ufd2a, E6.3.2.19), a ubiquitin assembly protein, and the full length of nicotinamide mononucleotide adenylyltransferase 1 (Nmnat1, E2.7.7.1), an enzyme that can directly catalyze the synthesis of nicotinamide adenine dinucleotide (NAD) [5], [6]. Both Nmnat1 activity and the short N-terminal were shown to have contributions to Wld^S^-mediated full axon protective effect [3], [7]. Interestingly, although Wld^S^ has very significant axon and synapse-protective function, it fails to protect the death of neuronal cell bodies induced by axotomy or deprivation of nerve growth factor [8], [9]. Until now, whether Wld^S^ can protect non-neuronal cells against toxic chemicals is still largely unknown.

Paraquat, a widely used and highly toxic bipyridylium herbicide, caused many fatalities by accidental or intentional ingestion [10]. It has been shown that oxidative stress and mitochondrial damage were involved in paraquat-induced toxicity [10]–[13]. Recent research has concentrated on the therapeutic potential of antioxidants, such as vitamin C, N-acetylcysteine, sodium salicylate, superoxide dismutase and its mimetic enzymes against paraquat-induced toxicity, but until now there hasn't been an effective antidote to be clinically applied for paraquat poisoning [10]. It has been shown that nicotinic acid, a precursor of NAD, could reduce paraquat-induced mortality in rats and prevent NAD decrease in the rat livers poisoned with paraquat [14]. Nicotinic acid was also reported to protect against paraquat-induced toxicity in bacteria and isolated perfused rat lung [15], [16]. Whether exogenously provided NAD or overexpression of NAD biosynthetic enzymes can reduce paraquat-induced cytotoxicity is still unclear.

NAD performs a variety of roles in the cell. By transferring electrons, NAD plays an important part in energy production by involving in the tricarboxylic acid cycle and the electron transport chain in mitochondria [17]. NAD also acts as a substrate for various enzymes including cADP-ribose synthases, poly (ADP-ribose) polymerase-1 (PARP-1) and the sirtuin family to be involved in the processes of cell signal transduction, DNA repair, gene transcription, and cell death [18]. NAD can be taken up from the extracellular surroundings, or can be synthesized either through de novo pathway, or through salvage pathway by recycling NAD derivatives such as nicotinic acid, nicotinamide, and nicotinamide riboside back to NAD [18]. Nicotinamide phosphoribosyltransferase (Nampt, E2.4.2.12) and Nmnat sequentially form a major salvage pathway to synthesize NAD from nicotinamide via the intermediate nicotinamide mononucleotide (NMN) in mammalian cells [19]. Knockdown or inhibition of Nampt can directly induce apoptosis, which can be reversed by exogenously provided NAD or its derivatives [20], [21]. Furthermore, providing sufficient extracellular NAD or overexpressing Nmnat1 or Nampt could replenish the decrease of intracellular NAD, and therefore conferred protection against cell death under various conditions [21]–[24]. These findings indicate that maintenance of intracellular NAD levels should be beneficial for cell survival.

SIRT1 is an NAD-dependent protein deacetylase of sirtuin family, and its activity is sensitively influenced by alteration of intracellular NAD concentration [18]. SIRT1 has been reported to be a key regulator of cell defense and survival in response to various kinds of stress such as DNA damage, oxidative stress, heat shock and ionizing radiation [25]–[27]. Consistently, previous report has shown that SIRT1 is a key link between NAD depletion and PARP-mediated cardiac myocyte cell death [23]. The synergic role of NAD and SIRT1 in cytotoxicity under different conditions is yet to be further elucidated.

In this study, we investigated whether Wld^S^ could confer protection to cytotoxicity induced by various toxic chemicals. We demonstrate that Wld^S^ reduces paraquat-induced cytotoxicity via an NAD dependent deacetylase SIRT1 by attenuating the depletion of NAD.

## Results

### Wld^S^ attenuates paraquat-induced cytotoxicity *in vitro* and *in vivo*

**Figure 1:**
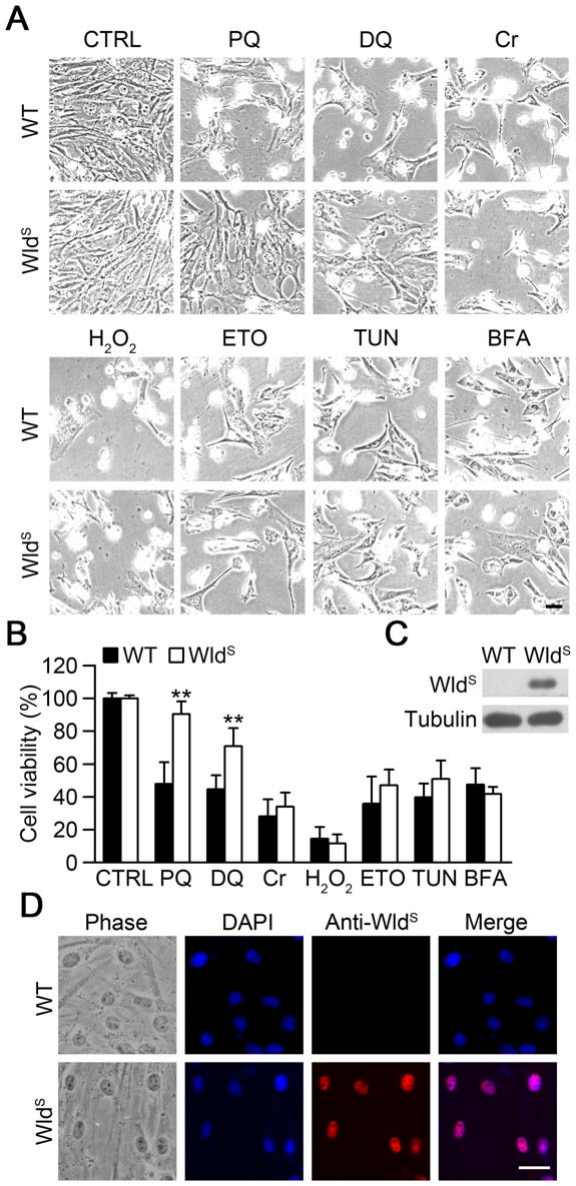
The susceptibility of Wld^S^ MEFs to cytotoxicity induced by various toxic chemicals. (A) Wld^S^ MEFs were resistant to cytotoxicity induced by paraquat and diquat. The representative images of MEFs prepared from wild-type (WT) or Wld^S^ mice were taken after treatment with 1 mM paraquat (PQ), 0.2 mM diquat (DQ), 20 µM potassium dichromate (Cr), 1 mM H_2_O_2_, 600 µM etoposide (ETO), 2 µg/ml tunicamycin (TUN) or 2 µg/ml brefeldin A (BFA) for 24 h. Scale bar, 40 µm. (B) Wld^S^ alleviated the loss of cell viability induced by paraquat and diquat. Cell viability was measured by MTT assay using the indicated MEFs with the same treatment as in (A) for 20 h. **p<0.01 versus WT treated with the same chemical, Student's t-test. In this and all other figures, error bars represent SD. (C) The expression of Wld^S^ protein in Wld^S^ MEFs was confirmed by western blot with anti-Wld^S^ antibody. Tubulin was measured as an internal control. (D) The nuclear localization of Wld^S^ protein in MEFs was measured by immunofluorescence using anti-Wld^S^ antibody. Nuclei were stained with DAPI. Scale bar, 20 µm.

To investigate whether Wld^S^ has cellular protective function, we used various toxic chemicals to treat mouse embryonic fibroblasts (MEFs) isolated from wild-type and Wld^S^ embryos. We observed that Wld^S^ MEFs attenuated the morphological changes induced by the bipyridylium herbicides paraquat and diquat compared with wild-type MEFs. However, Wld^S^ had no effect on the cytotoxicity induced by the oxidative stress inducers chromium (VI) and H_2_O_2_, the DNA damage inducer etoposide, and the endoplasmic reticulum stress inducers tunicamycin and brefeldin A (Figure 1A). Next we determined cell viability by MTT assay, which is based on the reduction of yellow tetrazolium salt MTT by mitochondrial dehydrogenases of viable cells to a blue-purple formazan that can be measured spectrophotometrically [28]. Consistently, we found Wld^S^ MEFs were significantly resistant to the cytotoxicity induced by paraquat and diquat (Figure 1B). Moreover, we measured the effect of Wld^S^ on MEFs treated with different concentration of H_2_O_2_ by MTT assay, and we further confirmed that Wld^S^ had no significant effect on the cytotoxicity induced by H_2_O_2_ (Figure S1). The expression of Wld^S^ in MEFs was confirmed by western blot (Figure 1C), and Wld^S^ protein localized in the nuclei of Wld^S^ MEFs when analyzed by immunofluorescence (Figure 1D). In addition, we found that Wld^S^ didn't alter the cell growth rate of MEFs and the expression levels of the cell stress marker phospho-histone H2A.X and the cell cycle progression marker acetyl-histone H3 in MEFs (Figure S2). This is consistent with the previous findings that expression of Wld^S^ protein has no adverse effects and induced no changes in cell cycle and cell stress status on non-neuronal tissues [29]. Taken together, these data show that Wld^S^ confers resistance to paraquat and diquat-induced cytotoxicity and has no obvious detrimental effect on MEFs.

**Figure 2:**
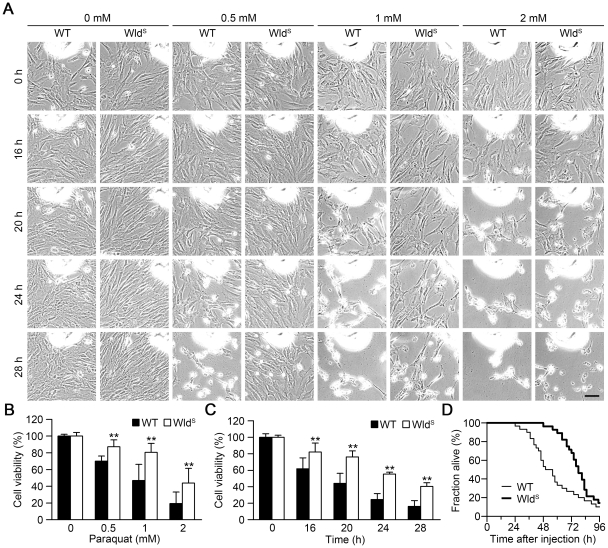
Wld^S^ attenuates paraquat-induced toxicity *in vitro* and *in vivo*. (A) The representative images of wild-type (WT) and Wld^S^ MEFs treated with different concentrations of paraquat were taken from the same field at the indicated times. Scale bar, 80 µm. (B and C) Wld^S^ alleviated the loss of cell viability induced by paraquat at the indicated concentrations for 20 h, or at 1 mM for the indicated times. Cell viability was determined by MTT assay. **p<0.01 versus WT with the same treatment, Student's t-test. (D) Wld^S^ slowed down the death of mice induced by intraperitoneal injection of paraquat (70 mg per kg body mass). The mice were monitored every 2 h for 96 h. Kaplan-Meier survival analysis showed significant difference between WT and Wld^S^ mice (p = 0.012, Cox's test; n = 30 for WT mice, n = 28 for Wld^S^ mice).

To further gain insight into the protective effect of Wld^S^ to paraquat-induced toxicity, we used different concentrations of paraquat to treat MEFs and took the representative images from the same field at the indicated times. As shown in Figure 2A, Wld^S^ significantly slowed down the process of morphological changes at each indicated dose of paraquat (Figure 2A). As shown in Figure 2B and 2C, Wld^S^ significantly alleviated the loss of cell viability induced by paraquat at the indicated concentrations for 20 h, or at 1 mM for the indicated times. Next, to further determine whether Wld^S^ has an *in vivo* protective effect against paraquat, we subjected adult wild-type and Wld^S^ mice to intraperitoneal injection with paraquat at a dose of 70 mg/kg. Wld^S^ mice resisted the challenge significantly longer than wild-type mice (Figure 2D). The median lethal time for the Wld^S^ mice was approximately 78 h, in contrast to about 53 h for the wild-type mice. These data clearly show that Wld^S^ has protective function against paraquat-induced toxicity both *in vitro* and *in vivo*.

### Wld^S^ delays paraquat-induced mitochondrial injury in MEFs

**Figure 3:**
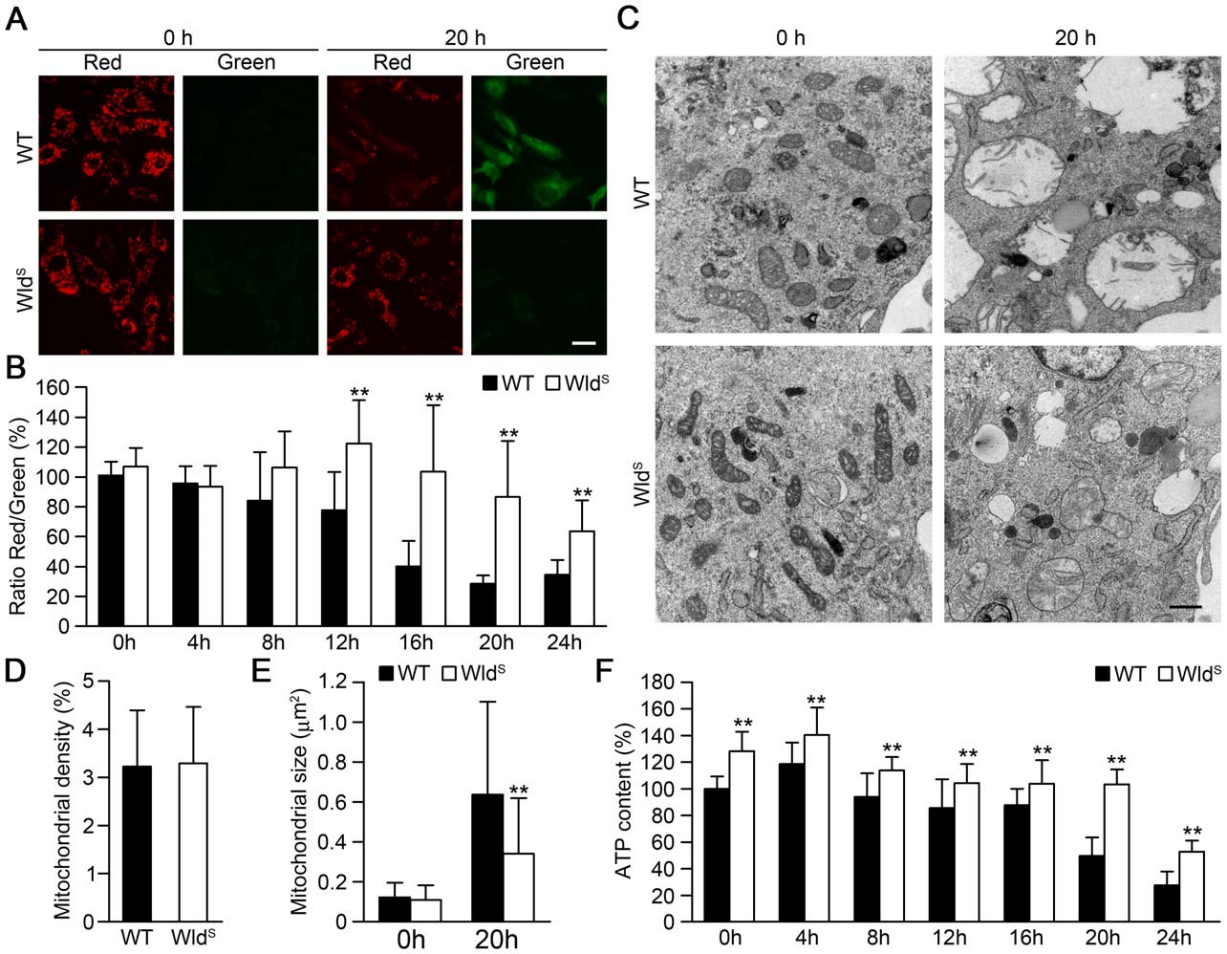
Wld^S^ delays paraquat-induced mitochondrial damage in MEFs. (A) Wld^S^ attenuated paraquat-induced disruption of mitochondrial membrane potential. The representative images of wild-type (WT) and Wld^S^ MEFs stained by mitochondrial membrane potential fluorescent indicator JC-1 after treatment with 1 mM paraquat for the indicated times. Scale bar, 20 µm. (B) The protective effect of Wld^S^ on paraquat-induced disruption of mitochondrial membrane potential in MEFs was assessed by the ratio of red to green fluorescence of JC-1. **p<0.01 versus WT with the same treatment, Student's t-test. (C) Wld^S^ attenuated the mitochondrial swelling and loss of regular cristae structure induced by paraquat. The representative transmission electron microscopy images of WT and Wld^S^ MEFs treated with 1 mM paraquat for the indicated times were shown. Scale bar, 0.5 µm. (D) and (E) Quantification of the mitochondrial density and mitochondrial size from (C). **p<0.01 versus WT with the same treatment, Student's t-test, n = 462 and 563 for WT and Wld^S^ respectively with 0-h treatment; n = 147 and 179 for WT and Wld^S^ respectively with 20-h treatment. (F) Wld^S^ slowed down the decrease of ATP levels in MEFs induced by 1 mM paraquat for the indicated times. **p<0.01 versus WT with the same treatment, Student's t-test.

To examine whether Wld^S^ could protect cells from paraquat-induced mitochondrial injury, we used JC-1 to detect the changes of the mitochondrial membrane potential. As shown in Figure 3A, an intact mitochondrial membrane potential visible in red, was seen in untreated wild-type and Wld^S^ MEFs. After exposure to 1 mM paraquat for 20 h, wild-type MEFs showed significant decrease of red fluorescence and profound increase of green fluorescence, indicating the collapse of mitochondrial membrane potential. In contrast, Wld^S^ MEFs did not show obvious change in their fluorescence pattern after the same treatment, indicating that Wld^S^ MEFs preserved the mitochondrial membrane potential. These results were further confirmed by quantification of the red-to-green fluorescence ratio (Figure 3B). Then we applied transmission electron microscopy to study the effect of Wld^S^ on mitochondrial morphology, density and size. As shown in Figure 3C-E, there was no obvious difference in mitochondrial morphology, density and size between untreated wild-type and Wld^S^ MEFs. However, after treatment with paraquat for 20 h, Wld^S^ MEFs showed much less severe mitochondrial swelling and loss of regular cristae structure compared with wild-type MEFs (Figure 3C and 3E). To further determine whether Wld^S^ could protect mitochondrial energetic function in paraquat-treated cells, we measured the ATP levels in wild-type and Wld^S^ MEFs. As shown in Figure 3F, ATP levels were slightly but significantly increased in Wld^S^ MEFs compared with wild-type MEFs, and Wld^S^ significantly slowed down the decrease of ATP levels induced by paraquat. After treatment with paraquat for 20 h, the ATP levels dropped to approximately 50% in wild-type MEFs, but the ATP levels still remained nearly unchanged in Wld^S^ MEFs (Figure 3F). As mitochondria were reported to be the major source of paraquat-induced reactive oxygen species (ROS) in the brain [30], we used CM-DCF-DA to measure intracellular H_2_O_2_ levels in MEFs after treatment with paraquat. Although paraquat significantly upregulated the intracellular H_2_O_2_ levels, Wld^S^ had no significant effect on intracellular H_2_O_2_ levels (Figure S3). Next, to determine whether Wld^S^ mitochondria are intrinsically more resistant to paraquat-induced injury, we isolated sufficient purified and functional mitochondria from the livers of wild-type and Wld^S^ mice. After the isolated liver mitochondria were treated with the indicated concentrations of paraquat, we found that Wld^S^ mitochondria showed no resistance to paraquat-induced disruption of membrane potential when measured by JC-1 staining (Figure S4B). In addition, we found Wld^S^ protein also localized in the nuclei of primary mouse hepatocytes (Figure S4A). Collectively, these data demonstrate that Wld^S^ delays paraquat-induced mitochondrial injury in MEFs.

### NAD synthesis activity of Wld^S^ is responsible for its protective function against the cytotoxicity induced by paraquat

**Figure 4:**
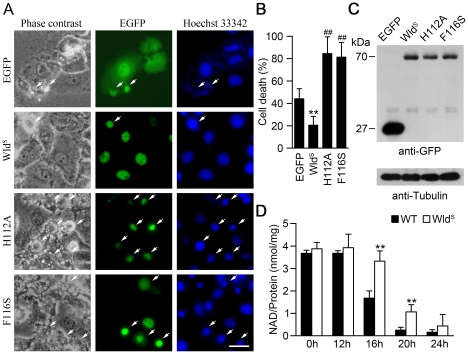
NAD synthesis activity of Wld^S^ is responsible for its protective effect on paraquat-induced cell death. (A) The NAD synthesis activity was required for the anti-apoptotic effect of Wld^S^. After transfection with the plasmids expressing EGFP, EGFP-Wld^S^ (Wld^S^), EGFP-Wld^S^H112A (H112A), or EGFP-Wld^S^F116S (F116S) for 24 h, the Hela cells were treated with paraquat for 24 h. Then the cells were stained with Hoechst 33342. Arrows indicate dead cells. Scale bar, 20 µm. (B) Quantification of the dead cells corresponding to (A). The percentage of dead cells was quantified in cells with green fluorescence. **p<0.01 compared with cells expressing EGFP, ##p<0.01 compared with cells expressing EGFP-Wld^S^, Student's t-test. (C) The expression of EGFP and EGFP-Wld^S^ and its mutants were confirmed by western blot with anti-EGFP. Tubulin was measured as an internal control. (D) The decrease of NAD levels was delayed in Wld^S^ MEFs treated with paraquat. Wild-type (WT) and Wld^S^ MEFs were treated with 1 mM paraquat for the indicated times to assess the NAD levels. **p<0.01 versus WT with the same treatment, Student's t-test.

It has been shown that NAD synthesis activity is important for Wld^S^ to exert its protective function both *in vitro* and *in vivo* [31]–[33]. To determine whether NAD synthesis activity of Wld^S^ is also responsible for the protective function against paraquat-induced cytotoxicity, we constructed plasmids expressing EGFP-fused Wld^S^ or enzyme-dead Wld^S^ with an H112A or F116S point mutation as previously described [7], [34]. As shown in Figure 4A and Figure 4B, Wld^S^ significantly protected Hela cells from paraquat-induced cell death, while enzyme-dead Wld^S^ disrupted the protective effect of Wld^S^, and even exhibited an opposite effect to promote cell death. The expression of EGFP-fused Wld^S^ and Wld^S^-H112A and Wld^S^-F116S protein were confirmed by western blot (Figure 4C). These results suggest that the protective effect of Wld^S^ requires its NAD synthesis activity. Next, we examined whether Wld^S^ could delay paraquat-induced NAD decrease. As shown in Figure 4D, intracellular NAD level was not significantly increased in Wld^S^ MEFs at basal level. However, Wld^S^ markedly delayed the NAD decline caused by paraquat (Figure 4D), which is similar to its protective effect on cell viability. These results imply that Wld^S^ could exert its protective effects via its NAD synthesis activity by attenuating NAD depletion after paraquat exposure.

### Exogenous NAD and NMN protect cells against the cytotoxicity and decrease of ATP and NAD levels induced by paraquat

**Figure 5:**
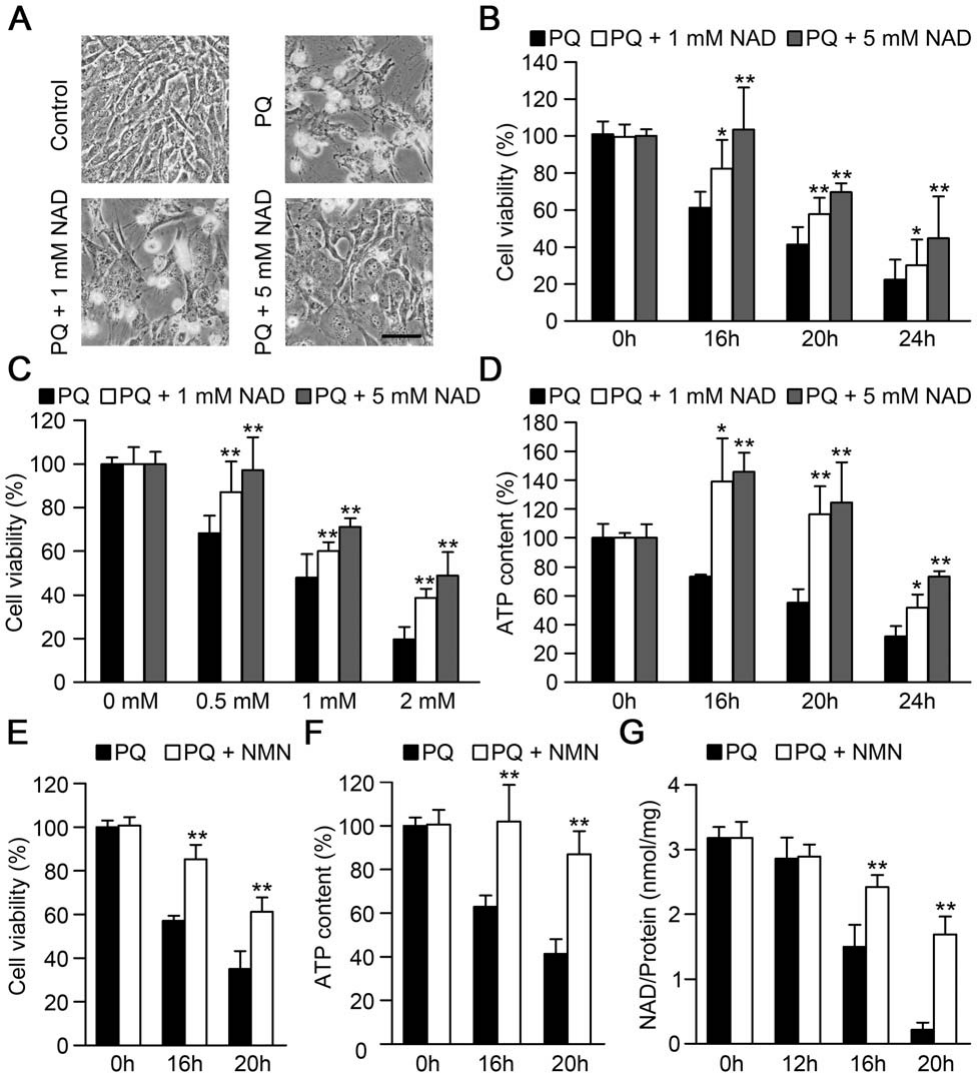
Exogenous NAD and NMN protect cells against paraquat-induced cytotoxicity and decline of ATP and NAD. (A) NAD protected MEFs against paraquat-induced cytotoxicity. Representative phase images were taken after the MEFs treated with or without 1 mM paraquat (PQ) and the indicated concentrations of NAD for 24 h. Scale bar, 40 µm. (B and C) NAD protected MEFs against the loss of cell viability induced by paraquat. Cell viability was measured by MTT assay with the MEFs treated with 1 mM paraquat and the indicated concentrations of NAD for the specified times (B), or treated with the indicated concentrations of paraquat and NAD for 20 h (C). *p<0.05, **p<0.01 versus the corresponding paraquat treatment without NAD, Student's t-test. (D) NAD delayed the decrease of ATP levels in MEFs treated with paraquat. MEFs were treated the same as in (B). *p<0.05, **p<0.01 versus the corresponding paraquat treatment without NAD, Student's t-test. (E) NMN protected MEFs from paraquat-induced cytotoxicity. MEFs were treated with 1 mM paraquat in the absence or presence of 1 mM NMN for the indicated times, and then MTT assay was performed to determine cell viability. **p<0.01 versus paraquat at the same time point, Student's t-test. (F and G) NMN delayed the decline of ATP and NAD levels induced by paraquat. MEFs were treated the same as in (E), and then the cells were harvested to measure the ATP and NAD levels respectively. **p<0.01 versus paraquat at the same time point, Student's t-test.

To further determine whether preventing the decline of intracellular NAD could result in protection against paraquat, we examined the protective effects of exogenous NAD. As shown in Figure 5A, NAD could significantly protect cells against paraquat-induced morphological changes. Similarly, MTT assays showed that NAD could alleviate the loss of cell viability induced by paraquat at different time points and doses (Figure 5B and 5C). Moreover, the decrease of cellular ATP levels induced by paraquat was also significantly attenuated by NAD (Figure 5D). To further confirm the protective role of NAD, we measured the effect of NMN, a precursor of NAD. As expected, we found that NMN also significantly protected cells against the cytotoxicity and decrease of ATP levels induced by paraquat (Figure 5E and 5F). Consistently, NMN delayed the decrease of intracellular NAD levels caused by paraquat (Figure 5G). All these results demonstrate that exogenous NAD and NMN can protect cells against the cytotoxicity and decrease of intracellular ATP and NAD levels induced by paraquat.

### SIRT1 is required for both Wld^S^ and exogenous NAD-mediated cellular protection against paraquat

**Figure 6:**
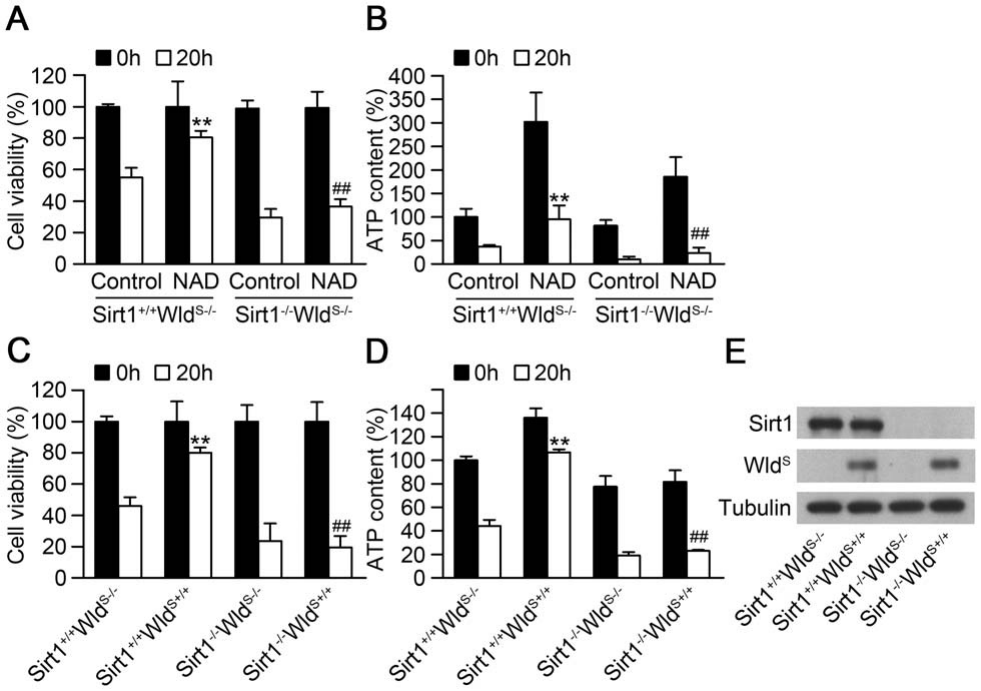
SIRT1 is required for both Wld^S^ and exogenous NAD-mediated cellular protection against paraquat. (A) Deletion of SIRT1 reduced the protective effect of exogenous NAD on cytotoxicity induced by paraquat. MEFs prepared from SIRT1^+/+^Wld^S-/-^ and SIRT1^-/-^Wld^S-/-^ embryos were treated with 1 mM paraquat in the absence or presence of 5 mM NAD for indicated times. Cell viability was then determined by MTT assay. **p<0.01 versus SIRT1^+/+^Wld^S-/-^ MEFs treated without NAD for 20 h, ##p<0.01 versus SIRT1^+/+^Wld^S-/-^ MEFs treated with NAD for 20 h, Student's t-test. (B) Exogenous NAD attenuated the decline of ATP levels caused by paraquat through SIRT1. MEFs were prepared and treated the same as in (A). Then the cells were collected to measure the ATP levels. **p<0.01 versus SIRT1^+/+^Wld^S-/-^ MEFs treated without NAD for 20 h, ##p<0.01 versus SIRT1^+/+^Wld^S-/-^ MEFs treated with NAD for 20 h, Student's t-test. (C) Wld^S^ reduced paraquat-induced cytotoxicity through SIRT1. MEFs prepared from SIRT1^+/+^Wld^S-/-^, SIRT1^+/+^Wld^S+/+^, SIRT1^-/-^Wld^S-/-^, SIRT1^-/-^Wld^S+/+^ embryos were treated with 1 mM paraquat for indicated times. Cell viability was then determined by MTT assay. **p<0.01 versus SIRT1^+/+^Wld^S-/-^ MEFs treated for 20 h. ##p<0.01 versus SIRT1^+/+^Wld^S+/+^ MEFs treated for 20 h, Student's t-test. (D) Wld^S^ attenuated the decrease of ATP levels caused by paraquat through SIRT1. MEFs were prepared and treated the same as in (C). Then the cells were collected to measure the ATP levels. **p<0.01 versus SIRT1^+/+^Wld^S-/-^ MEFs treated for 20 h, ##p<0.01 versus SIRT1^+/+^Wld^S+/+^ MEFs treated for 20 h, Student's t-test. (E) The expression of SIRT1 and Wld^S^ were confirmed by western blot with anti-SIRT1 or anti-Wld^S^ antibody. Tubulin was measured as an internal control.

To examine whether SIRT1 is involved in Wld^S^ and/or exogenous NAD-mediated protection against paraquat-induced cytotoxicity, we used MEFs prepared from SIRT1^+/+^Wld^S-/-^, SIRT1^+/+^Wld^S+/+^, SIRT1^-/-^Wld^S-/-^, or SIRT1^-/-^Wld^S+/+^ embryos, and the expression of SIRT1 and Wld^S^ in different MEFs were confirmed by western blot (Figure 6E). From the results of MTT assay, we found that the protective effect of exogenous NAD was significantly blocked in SIRT1^-/-^Wld^S-/-^ MEFs (Figure 6A). Moreover, exogenous NAD failed to delay the decrease of ATP levels in SIRT1^-/-^Wld^S-/-^ MEFs (Figure 6B). Similarly, Wld^S^ failed to attenuate the cytotoxicity of SIRT1^-/-^Wld^S+/+^ MEFs induced by paraquat (Figure 6C). The decline of ATP levels induced by paraquat was also not attenuated in SIRT1^-/-^Wld^S+/+^ MEFs (Figure 6D). These results indicate that SIRT1 is required for both Wld^S^ and exogenous NAD-mediated cellular protection against paraquat.

## Discussion

In this study, we demonstrate that Wld^S^ attenuates the toxicity induced by the herbicide paraquat *in vitro* and *in vivo*. Wld^S^ has been shown to protect axons and synapses against various injuries including mechanical, genetic and chemical insults [1], [3], [4], [7], but shows no effect on the apoptosis of neuronal cell body induced by axotomy or deprivation of nerve growth factor [8], [9]. Similarly, we found that Wld^S^ did not provide cellular protection against the inducers of oxidative stress, DNA damage and endoplasmic reticulum stress, but Wld^S^ showed significant protective function against cytotoxicity induced by paraquat and diquat. Oxidative stress and mitochondrial damage have been shown to be involved in paraquat-induced cytotoxicity [10]–[13]. Here, we found that Wld^S^ failed to alleviate ROS upregulation induced by paraquat, which is consistent with our observation that Wld^S^ could not protect the cytotoxicity induced by the oxidative stress inducer chromium (VI) or hydrogen peroxide. Previous report has shown that a mitochondria-localized Nmnat isoform Nmnat3 could reduce oxidative or mitochondrial stress-induced ROS generation to delay axonal degeneration [35]. We found that Wld^S^ protein localized in the nuclei of Wld^S^ MEFs and primary mouse hepatocytes. The different intracellular localization of Nmnat3 and Wld^S^ might contribute to their different effects on intracellular ROS levels. Furthermore, we found Wld^S^ significantly delayed paraquat-induced mitochondrial injury in MEFs. Meanwhile, similar disruption of membrane potential was observed in purified liver mitochondria from wild-type and Wld^S^ mice after paraquat treatment, suggesting that mitochondria from Wld^S^ mice are not intrinsically more resistant to paraquat-induced injury. Previous reports showed that Wld^S^ might modify mitochondrial function by altering the expression of some genes involved in regulating mitochondrial stability and degeneration or by inhibiting the activation of mitochondrial permeability transition pore [36], [37], which might be part of the mechanisms involved in the protective function of Wld^S^ against paraquat-induced mitochondrial injury. It has been suggested that axonal protective function of Wld^S^ is mediated by its effect on bioenergetics [38]. Similarly, we found that Wld^S^ MEFs have increased ATP content at basal level compared with wild-type MEFs, and Wld^S^ significantly delays the decrease of ATP induced by paraquat. These results implicate that Wld^S^ might also exert cellular protective function through its bioenergetic role.

Furthermore, we found that NAD synthesis activity of Wld^S^ is essential for its protective function against cytotoxicity induced by paraquat. Similarly, the activity of Wld^S^ to synthesize NAD has been reported to be responsible for its axon sparing ability [33]. It has also been reported that Wld^S^ exerts its axon-protective function by compensating for the fast proteasome-dependent degradation of Nmnat2, an important enzyme in NAD synthesis [39]. In this study, we found that the proteasome inhibitor MG-132 had no effect on paraquat-induced cytotoxicity and Wld^S^-mediated protection in MEFs (Figure S5), suggesting that proteasome-dependent degradation of Nmnat2 is not involved in Wld^S^-mediated protection against paraquat. Previous studies have shown that NAD levels are not upregulated in the brain of Wld^S^ mice or axons of dorsal root ganglia overexpressing Wld^S^, but Wld^S^ delays the decline of NAD levels in degenerating axons [5], [38]. Consistently, we found that Wld^S^ didn't upregulate the NAD levels in MEFs, but significantly attenuated the decline of NAD levels induced by paraquat. NMN can be directly converted to NAD by Wld^S^ or Nmnats [5], [18]. We found, treatment with NAD or NMN also protected cells against cytotoxicity and decline of NAD levels induced by paraquat (Figure 5). These findings suggest the protective role of intracellular NAD against paraquat. Consistently, upregulation or replenishment of intracellular NAD has been reported to promote cell survival under various conditions [21]–[24]. Further studies focused on NAD synthesis pathway will provide more information about how NAD level is regulated to maintain cell survival.

NAD is an essential substrate for various enzymes such as SIRT1 to exert their functions [18]. The activity of NAD-dependent deacetylase SIRT1 has been shown to be regulated by NAD biosynthesis via Nampt in various biological processes [40]–[42]. In addition, NAD depletion induced by PARP activation reduced SIRT1 deacetylase activity, contributing to myocyte cell death during heart failure [23]. Similarly, in the present study, we found that SIRT1 was required for the protective effects of both Wld^S^ and exogenous NAD on cytotoxicity and ATP decrease induced by paraquat. SIRT1 has been well demonstrated to promote cell survival under various kinds of stress through deacetylating DNA repair factor Ku70 or transcription factors including p53, the FOXO family and heat shock factor [25], [27], [43], [44]. Further studies on the downstream effectors of SIRT1 will provide more insight into the effect of Wld^S^ and exogenous NAD in attenuating cytotoxicity. In conclusion, we demonstrated that Wld^S^ could confer resistance to paraquat both *in vitro* and *in vivo*. Similarly, exogenous NAD and NMN are also capable of reducing paraquat-induced cytotoxicity. Intracellular NAD and its effector SIRT1 are responsible for the protective function of Wld^S^. These findings provide new clues for the mechanisms underlying the protective function of Wld^S^, and imply that therapeutic strategies directed at maintenance of intracellular NAD level may be valuable for treating paraquat poisoning.

## Materials and Methods

### Ethics statement

All animal experimental procedures were approved by the Institutional Animal Care and Use Committee of the Institute for Nutritional Sciences (Protocol number 2007-AN-9).

### Materials

Paraquat, diquat, potassium dichromate (chromium (VI)), etoposide, tunicamycin, brefeldin A, β-nicotinamide mononucleotide (NMN) and 3-[4,5-dimethylthiazol-2-yl]-2,5-diphenyl-tetrazolium bromide (MTT) were purchased from Sigma. Hydrogen peroxide was from Merck. 5,5′,6,6′-tetrachloro-1,1′,3,3′-tetraethylbenzimida-zolylcarbocyanine iodide (JC-1) was from Molecular Probes. Nicotinamide adenine dinucleotide (NAD) was from Roche.

### Animals

Male C57BL/6 mice at 10 weeks of age were purchased from Slac (Shanghai, China). C57BL/Wld^S^ (Wld^S^) mice were purchased from Harlan Olac (Bicester, UK). SIRT1^+/−^ mice were a kind gift from Dr. Michael McBurney, University of Ottawa, Canada. Genotyping for Wld^S^ and SIRT1 was performed as described previously [45], [46].

### Preparation of mouse embryonic fibroblasts (MEFs)

Wld^S^ mice were mated with SIRT1^+/−^ mice to obtain Wld^S+/−^SIRT1^+/−^ mice, which were intercrossed to generate SIRT1^+/+^Wld^S-/-^, SIRT1^+/+^Wld^S+/+^, SIRT1^-/-^Wld^S-/-^, SIRT1^-/-^Wld^S+/+^ embryos. MEFs were prepared from the embryos with the indicated genotypes as previously described with minor modifications [47]. Briefly, individual embryo from the pregnant mice 14 to 16 days postcoitus was released into PBS, then head and internal organs were removed, and the trunk was minced and stirred in 0.25% trypsin for 5 min. The cell suspension was then cultured in DMEM containing 10% FBS, 100 U/ml penicillin and 0.1 mg/ml streptomycin. After 2 h, the adherent MEFs were washed twice with PBS, and maintained in the culture medium again. At least three embryos were used for each genotype. MEFs from passage 2 to 4 were used for this study.

### MTT assay

MEFs were seeded in 24-well plates at a density of 10^5^ cells per well, and incubated overnight. After treatment with or without paraquat and/or the indicated chemicals for the specified times, the medium was replaced with fresh culture medium containing 0.5 mg/ml MTT. After 4 h incubation, the medium was carefully aspirated and DMSO was added to dissolve crystals. Absorbance was measured at 570 nm with a SpectraMax 190 microplate reader (Molecular Devices).

### Immuno fluorescence

The cells were fixed with 4% paraformaldehyde for about 40 min at 4°C, and permeabilized and blocked with PBS containing 0.1% Triton X-100 and 3% BSA. After incubation with rabbit anti-Wld^S^ antibody (1∶400, a gift from Dr. Michael Coleman, Babraham Institute, UK) [32], the cells were stained with anti-rabbit antibody conjugated with Alexa Fluor 555 (1∶1000, Molecular Probes) in the dark for 1 h. Then the cells were stained with DAPI (0.5 µg/ml) and observed under a fluorescence microscope.

### Western blot

The cultured cells were directly harvested in SDS-PAGE loading buffer. Total cellular proteins were then analyzed by anti-Wld^S^ (1∶2000), anti-tubulin (1∶10000, Sigma), anti-GFP (1∶1000, Cell Signaling Technology), anti-SIRT1 antibody (1∶2000, Upstate Biotechnology), anti-phospho-histone H2A.X (Ser139) (1∶500, Upstate Biotechnologies) or anti-acetyl-histone H3 (Lys9) (1∶500, Cell Signaling). The immune complexes were detected using horseradish peroxidase-conjugated secondary antibodies including anti-rabbit IgG (1∶2000, Jackson ImmunoResearch) and anti-mouse IgG (1∶5000, Jackson ImmunoResearch) and visualized with chemiluminescence reagent (Pierce).

### Survival analysis

Survival analysis was performed by intraperitoneal injection of paraquat (70 mg per kg body mass) to 30 male C57BL/6 mice and 28 male Wld^S^ mice at 11 weeks of age. Then the mice were monitored every 2 h for 96 h.

### Mitochondrial membrane potential staining in MEFs

Mitochondrial membrane potential was assessed by JC-1 dye as previously described with minor modifications [48]. JC-1 can selectively enter into mitochondria. In normal mitochondria, JC-1 forms aggregates that can emit red fluorescence. When the mitochondrial membrane potential was disrupted, JC-1 dye leaks into the cytoplasm and can emit green fluorescence as monomers. The calculated ratio between the red and green fluorescence is proportional to the mitochondrial membrane potential. MEFs were seeded in 6-well plates at a density of 4×10^5^ cells per well, then incubated overnight. After treatment with paraquat for the indicated times, the cells were incubated in DMEM containing 5 µg/ml JC-1 dye for 20 min at 37°C in the dark. After washing twice, representative images were taken by a fluorescence microscope, or the red and green fluorescence of the cells was measured using a fluorescence plate reader (Flexstation II 384, Molecular Devices) to quantify the protective effect of Wld^S^ on mitochondrial membrane potential.

### Electron microscopy

The MEFs were trypsinized and washed twice with PBS. Then the cells were fixed in suspension with 2.5% glutaraldehyde and postfixed with 1% osmium tetroxide. Subsequently, the cells were dehydrated, embedded and solidified according to the usual methods. The ultra-thin sections were stained with 3% uranyl acetate, followed by lead citrate staining and examined using a JEOL JEM-1230 transmission electron microscope (JEOL, Peabody, MA). Micrographs of randomly selected areas and whole cells were obtained at a magnification of 20000× and 6000× respectively. Mitochondrial size and cell area were measured using Image-Pro Plus software. Mitochondrial density was measured with at least fifteen individual cells for each genotype, and was expressed as the percentage of cell area occupied by mitochondria.

### Measurement of ATP levels

ATP levels were measured using ATP bioluminescent somatic cell assay kit (Sigma) according to the manufacturer's instructions.

### Plasmids

pCMV-Wld^S^ and pCMV-Wld^S^F116S with F28S mutation in Nmnat1 were obtained as previously described [34]. pCMV-Wld^S^H112A was engineered from pCMV-Wld^S^ with Quick Change II site-directed mutagenesis kit (Stratagene, USA) using primers CCCCATCACCAACATGGCCCTCAGGCTGTTCGAG and CTCGAACAGCCTGAGGGCCATGTTGGTGATGGGG. To construct plasmids expressing EGFP-fused proteins, the cDNAs coding for Wld^S^, Wld^S^H112A and Wld^S^F116S were subcloned from pCMV-Wld^S^, pCMV-Wld^S^H112A and pCMV-Wld^S^F116S into pEGFP-C3 (Clontech) by PCR using the primers CGCGAATTCTGATGGAGGAGCTGAGCACTGACG and CGCGGATCCTCACAGAGTGGAATGG.

### Cell death analysis

Hela cells obtained from Cell Bank of the Chinese Academy of Science were cultured in DMEM containing 10% FBS, 100 U/ml penicillin and 0.1 mg/ml streptomycin, and transfected with 0.8 µg pEGFP-C3, pEGFP-Wld^S^, pEGFP-Wld^S^H112A, or pEGFP-Wld^S^F116S per well in 24-well plates using Lipofectamine 2000 (Invitrogen) according to the manufacturer's instructions. After transfection for 24 h, the cells were treated with 0.2 mM paraquat for 24 h. Then the cells were stained with 1 µg/ml Hoechst 33342 (Sigma), and observed under a fluorescence microscope. Dead EGFP positive cells were determined according to their morphological changes and condensed or fragmented nuclei as previously described [49].

### NAD assay

NAD was measured by the recycling assay as previously described [50] with minor modifications. Cells were extracted with 0.5 M HClO_4_ and then neutralized with 1 M KOH prepared in 125 mM Gly-Gly buffer (pH 7.4). Subsequently the samples were centrifuged at 10,000 g for 5 min. 20 µl supernatant was mixed with 100 µl freshly prepared reaction solution containing 0.2 mM MTT, 0.8 mM phenazine methosulfate, 9 units/ml alcohol dehydrogenase (Sigma), 67 mM nicotinamide, and 1.9% ethanol in 63 mM Gly-Gly buffer (pH 7.4). After incubation at 37°C for 60 min, 100 µl 0.1 M HCl in anhydrous isopropanol was added to dissolve the formazan crystals. Then absorbance at 560 nm was determined, and results were calibrated with NAD standards. Results were normalized to protein concentration as determined by BCA protein assay kit (Thermo).

### Measurement of intracellular H_2_O_2_ levels

Intracellular H_2_O_2_ levels was measured by flow cytometry after staining MEFs with CM-DCF-DA. The detailed procedure is described in the supplemental methods (Methods S1).

### Isolation and culture of primary mouse hepatocytes

Primary mouse hepatocytes were isolated from 12-week-old C57BL/6 mice and Wld^S^ mice. The detailed isolation method and culture condition are described in the supplemental methods (Methods S1).

### Measurement of the isolated mitochondrial membrane potential

Mitochondria were isolated from livers of wild-type and Wld^S^ mice and stained with JC-1. JC-1 red fluorescence was then measured to assess mitochondrial membrane potential. The detailed procedure is described in the supplemental methods (Methods S1).

### Statistics

Data are expressed as mean ± SD of at least three independent experiments. For group comparisons, we used Student's t-test. The significance of survival curves was determined by Cox's test. Differences were considered to be statistically significant when p<0.05.

## Supporting Information

Figure S1 **Wld^S^ can not alleviate the loss of cell viability induced by H_2_O_2_ in MEFs.** Wild-type (WT) and Wld^S^ MEFs were treated with the indicated concentrations of H_2_O_2_ for 20 h, and then cell viability was determined by MTT assay.
(TIF)

Figure S2 **Wld^S^ has no significant detrimental effect in MEFs.** (A) Wld^S^ MEFs had the same growth rate as wild-type (WT) MEFs. MEFs were plated in 24-well plates at a density of 5×10^4^ cells per well, then cell proliferation was determined by MTT assay at the indicated time points. (B) Wld^S^ didn't change the levels of cell stress protein phospho-histone H2A.X and cell cycle protein acetyl-histone H3 in MEFs. Cell lysates from WT and Wld^S^ MEFs were analyzed by western blot. Tubulin was measured as an internal control. (C) Quantification of phospho-histone H2A.X and acetyl-histone H3 protein levels corresponding to (B).
(TIF)

Figure S3 **Wld^S^ doesn't attenuate paraquat-induced ROS production in MEFs.** (A) Paraquat-induced intracellular H_2_O_2_ levels in Wld^S^ MEFs were similar to those in wild-type (WT) MEFs. After treatment with or without 1 mM paraquat for 20 h, the intracellular levels of H_2_O_2_ in MEF cells were measured with CM-DCF-DA by flow cytometry analysis. (B) Quantification of the fluorescence intensity corresponding to (A). **p<0.01 versus wild-type MEFs in control group, ##p<0.01 versus Wld^S^ MEFs in control group, Student's t-test.
(TIF)

Figure S4 **Liver mitochondria from Wld^S^ mice are as sensitive as wild-type to paraquat-induced membrane potential disruption.** (A) The nuclear localization of Wld^S^ protein in primary mouse hepatocytes was measured by immunofluorescence using anti-Wld^S^ antibody. Nuclei were stained with DAPI. Scale bar, 20 µm. (B) Isolated Wld^S^ mitochondria were as sensitive as wild-type (WT) to paraquat-induced membrane potential disruption. After treatment with the indicated concentrations of paraquat, membrane potential of isolated liver mitochondria from WT and Wld^S^ mice was measured by JC-1 staining.
(TIF)

Figure S5 **Paraquat-induced MEF cell death is not a proteasome-dependent process.** Wild-type (WT) and Wld^S^ MEFs were pretreated with the indicated concentrations of MG-132 for 3 h, then cotreated with 1 mM paraquat for 20 h. Subsequently, cell viability was determined by MTT assay. **p<0.01 versus WT with the same treatment, Student's t-test.
(TIF)

Methods S1 Supplemental Methods.
(DOC)
