# Supplementary figures and images for: WldS Reduces Paraquat-Induced Cytotoxicity via SIRT1 in Non-Neuronal Cells by Attenuating the Depletion of NAD
Source: PLoS One. 2011 Jul 5;6(7):e21770. doi: 10.1371/journal.pone.0021770 (PMC3130051; doi:10.1371/journal.pone.0021770)

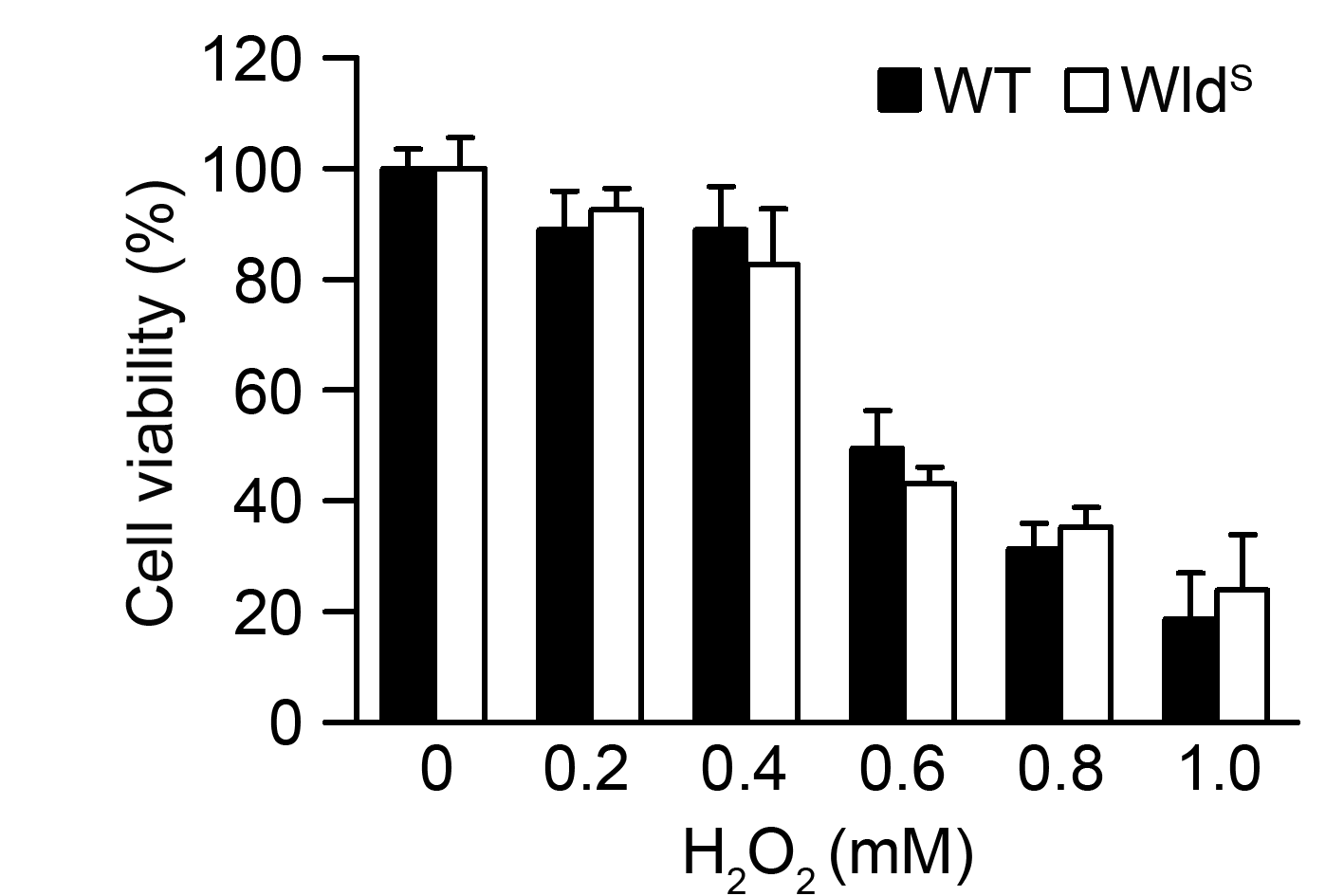

Supplement: Figure S1 — WldS can not alleviate the loss of cell viability induced by H2O2 in MEFs. Wild-type (WT) and WldS MEFs were treated with the indicated concentrations of H2O2 for 20 h, and then cell viability was determined by MTT assay. (TIF) [file pone.0021770.s001.tif]

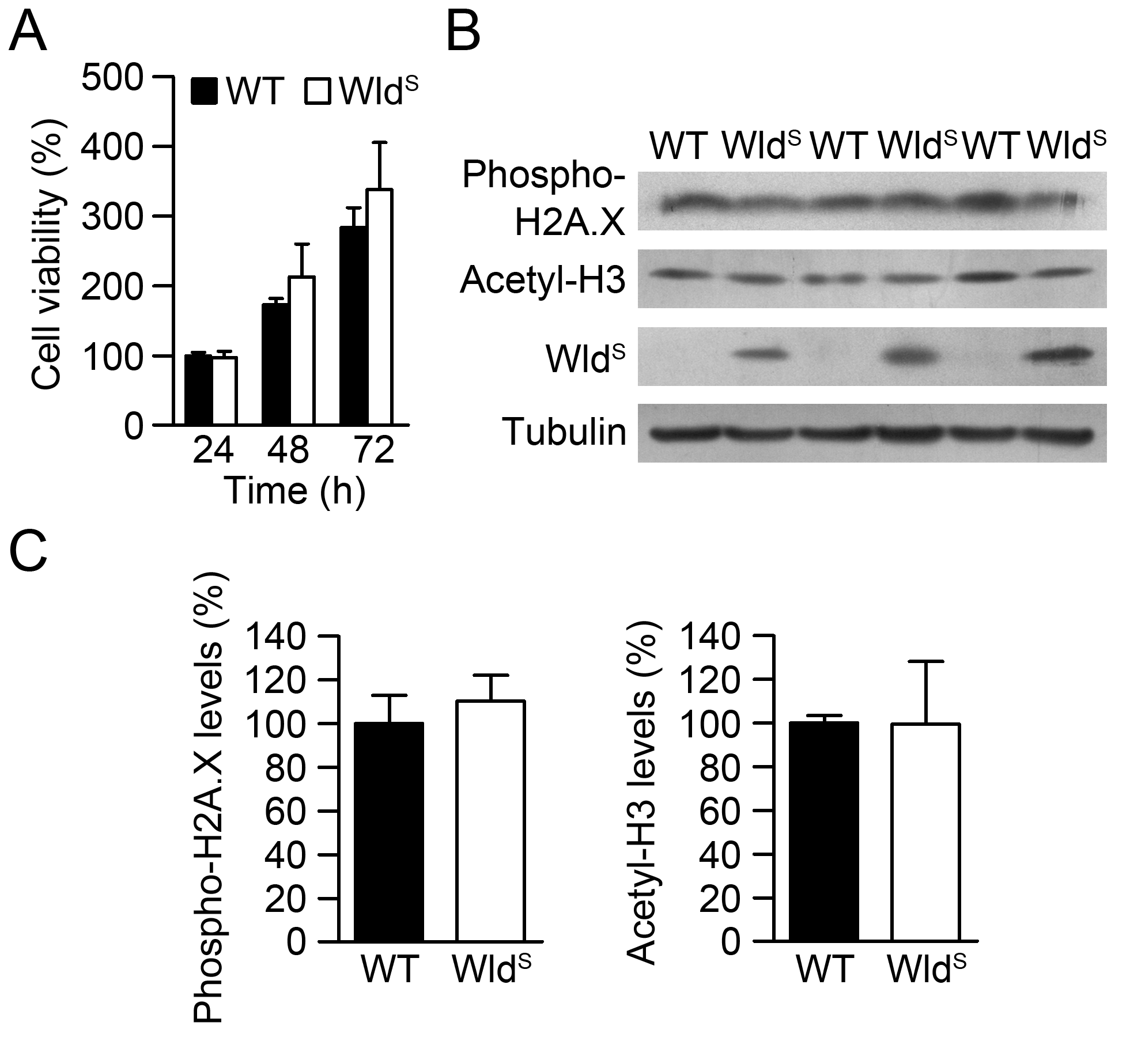

Supplement: Figure S2 — WldS has no significant detrimental effect in MEFs. (A) WldS MEFs had the same growth rate as wild-type (WT) MEFs. MEFs were plated in 24-well plates at a density of 5×10^4^ cells per well, then cell proliferation was determined by MTT assay at the indicated time points. (B) WldS didn't change the levels of cell stress protein phospho-histone H2A.X and cell cycle protein acetyl-histone H3 in MEFs. Cell lysates from WT and WldS MEFs were analyzed by western blot. Tubulin was measured as an internal control. (C) Quantification of phospho-histone H2A.X and acetyl-histone H3 protein levels corresponding to (B). (TIF) [file pone.0021770.s002.tif]

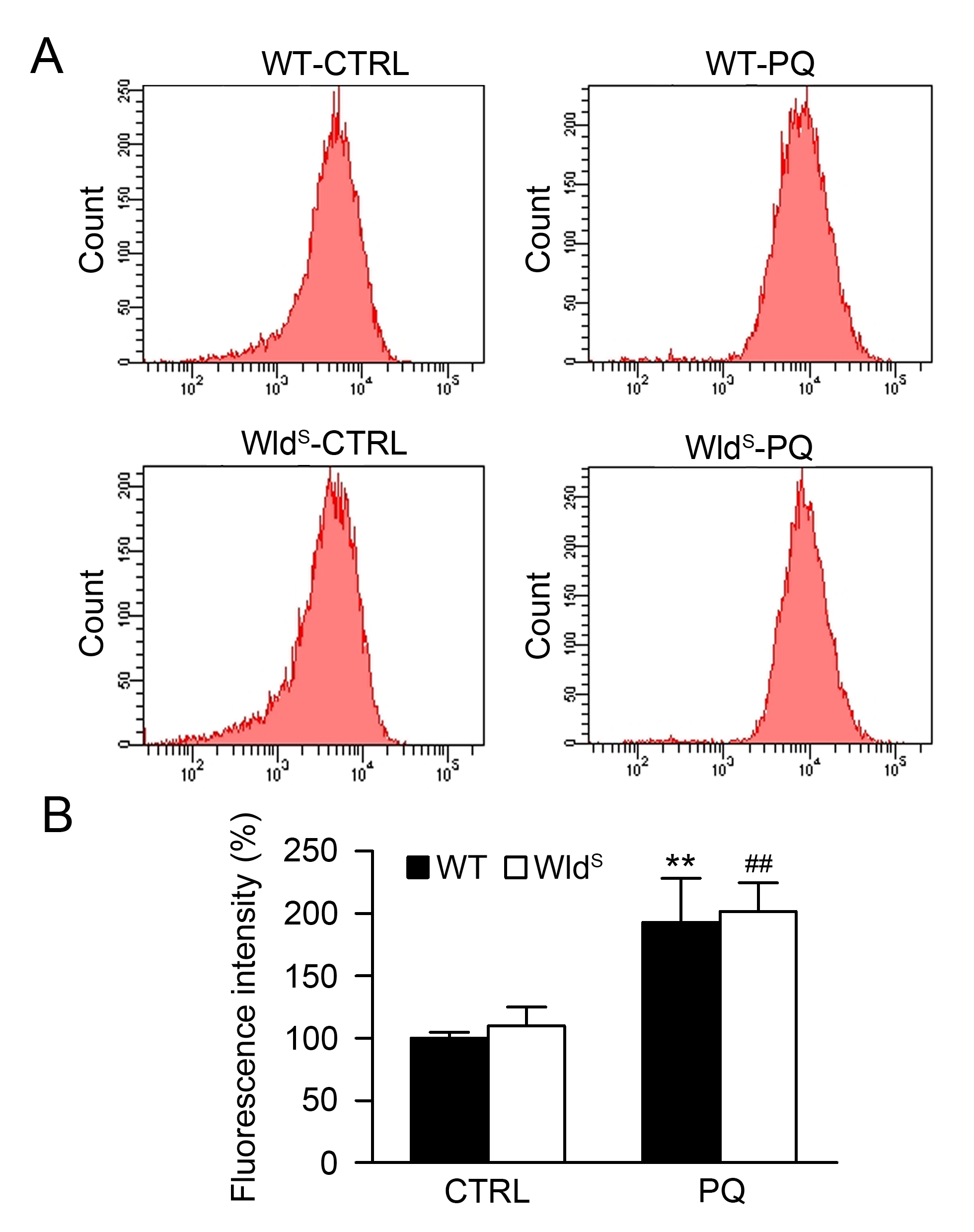

Supplement: Figure S3 — WldS doesn't attenuate paraquat-induced ROS production in MEFs. (A) Paraquat-induced intracellular H2O2 levels in WldS MEFs were similar to those in wild-type (WT) MEFs. After treatment with or without 1 mM paraquat for 20 h, the intracellular levels of H2O2 in MEF cells were measured with CM-DCF-DA by flow cytometry analysis. (B) Quantification of the fluorescence intensity corresponding to (A). **p<0.01 versus wild-type MEFs in control group, ##p<0.01 versus WldS MEFs in control group, Student's t-test. (TIF) [file pone.0021770.s003.tif]

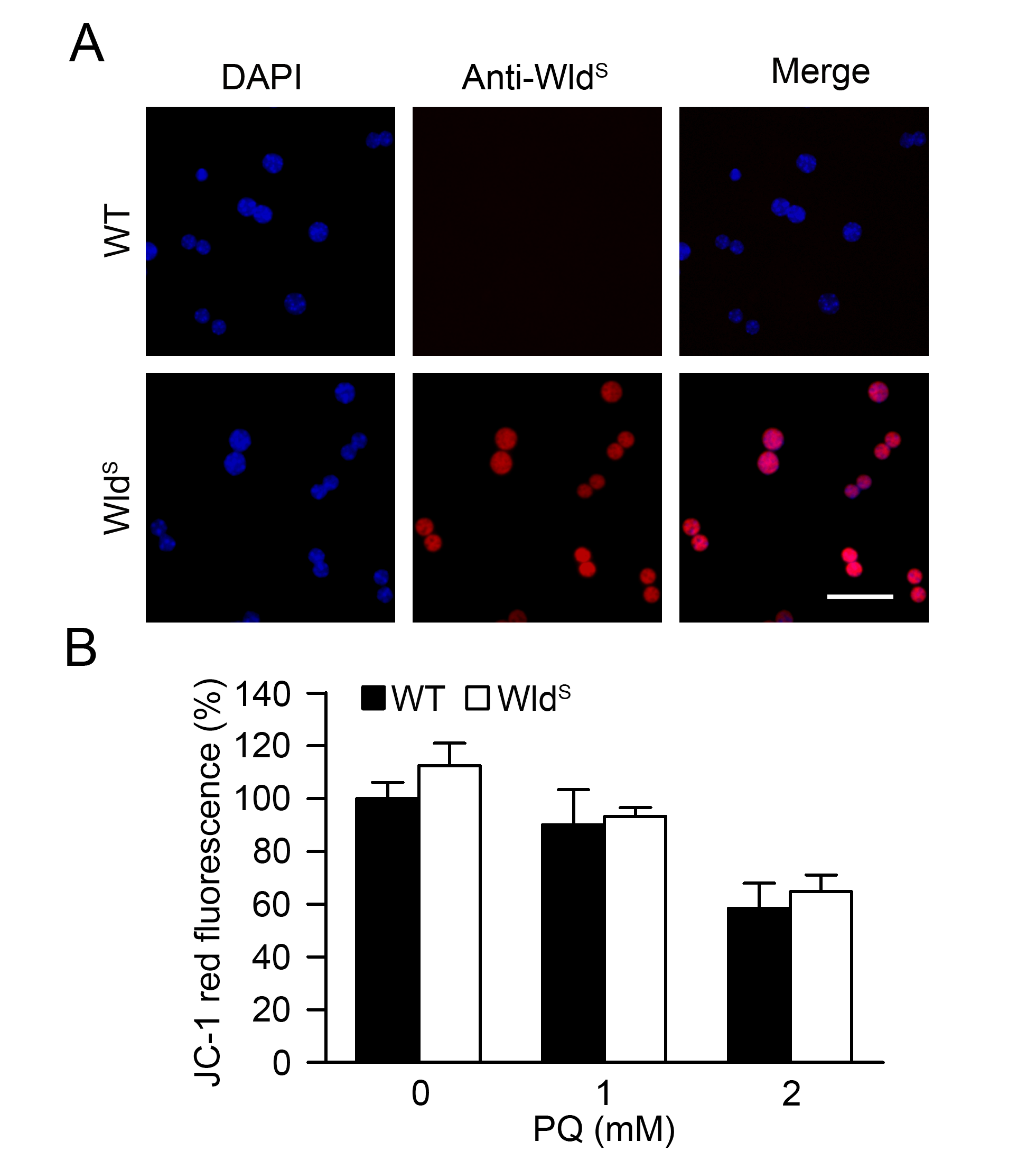

Supplement: Figure S4 — Liver mitochondria from WldS mice are as sensitive as wild-type to paraquat-induced membrane potential disruption. (A) The nuclear localization of WldS protein in primary mouse hepatocytes was measured by immunofluorescence using anti-WldS antibody. Nuclei were stained with DAPI. Scale bar, 20 µm. (B) Isolated WldS mitochondria were as sensitive as wild-type (WT) to paraquat-induced membrane potential disruption. After treatment with the indicated concentrations of paraquat, membrane potential of isolated liver mitochondria from WT and WldS mice was measured by JC-1 staining. (TIF) [file pone.0021770.s004.tif]

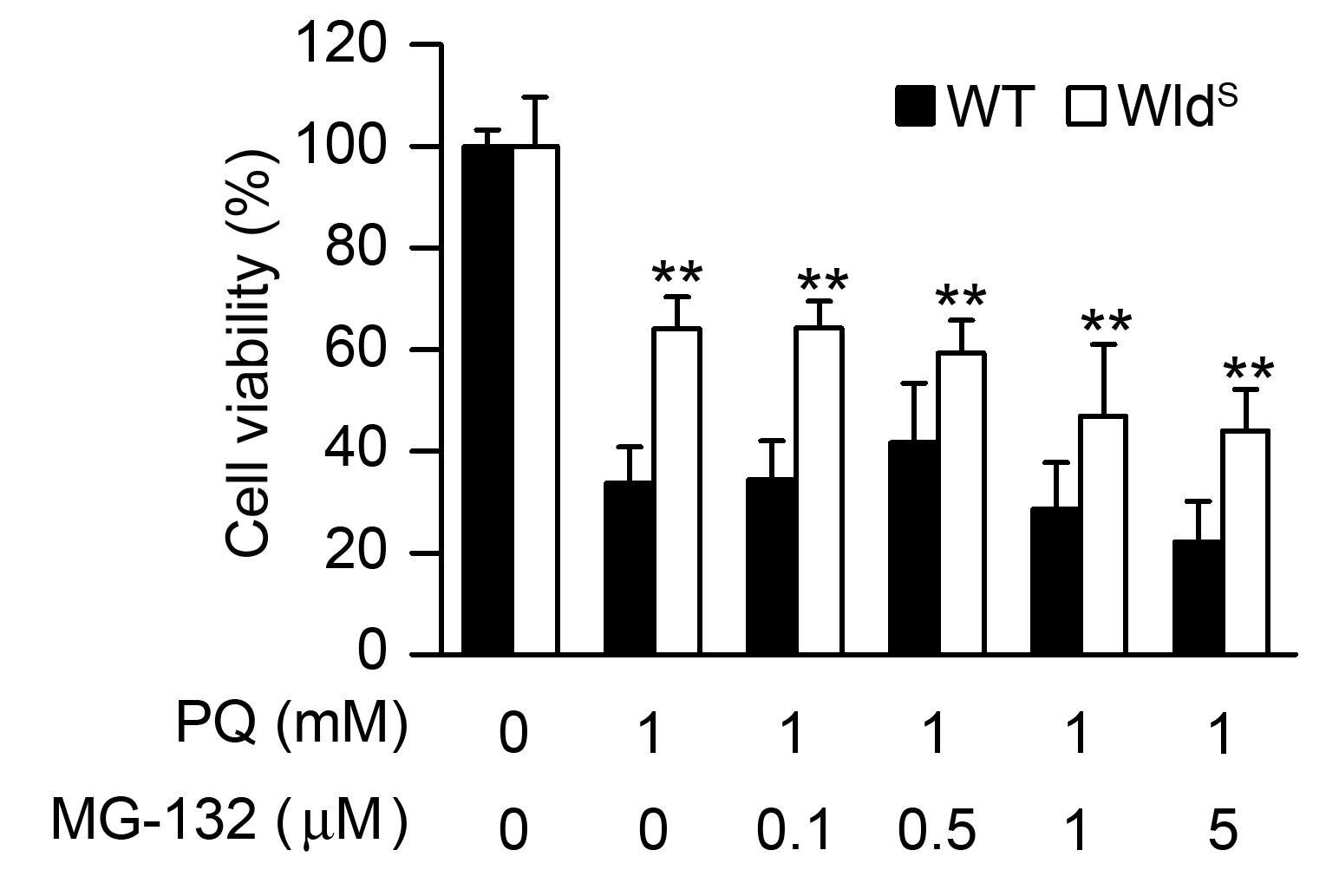

Supplement: Figure S5 — Paraquat-induced MEF cell death is not a proteasome-dependent process. Wild-type (WT) and WldS MEFs were pretreated with the indicated concentrations of MG-132 for 3 h, then cotreated with 1 mM paraquat for 20 h. Subsequently, cell viability was determined by MTT assay. **p<0.01 versus WT with the same treatment, Student's t-test. (TIF) [file pone.0021770.s005.tif]
