# Supplementary material for: WldS Reduces Paraquat-Induced Cytotoxicity via SIRT1 in Non-Neuronal Cells by Attenuating the Depletion of NAD
Source: PLoS One. 2011 Jul 5;6(7):e21770. doi: 10.1371/journal.pone.0021770 (PMC3130051; doi:10.1371/journal.pone.0021770)
Supplement: Methods S1 — Supplemental Methods. (DOC) [file pone.0021770.s006.doc]

**Wld^S^ Reduces Paraquat-induced Cytotoxicity via SIRT1 in Non-neuronal Cells by Attenuating the Depletion of NAD**

Qiujing Yu^1^, Ting Wang^1^, Xuexia Zhou^1^, Jingxia Wu^1^, Xingmiao Chen^1^, Yang Liu^1^, Dongmei Wu^1^, Qiwei Zhai^1*^

^1^Key Laboratory of Nutrition and Metabolism, Institute for Nutritional Sciences, Shanghai Institutes for Biological Sciences, Chinese Academy of Sciences; Graduate School of the Chinese Academy of Sciences; Shanghai, China

* E-mail: [qwzhai@sibs.ac.cn](mailto:qwzhai@sibs.ac.cn)

**Supplemental Methods**

Measurement of intracellular H_2_O_2_ levels

Intracellular H_2_O_2_ levels were measured as previously described with minor modifications [1]. MEFs were seeded in 6-well plates at a density of 4 × 10^5^ cells per well, and incubated overnight. After MEFs were treated with or without paraquat for 20 h, CM-DCF-DA (Molecular Probes) was added into the culture medium to a final concentration of 5 µM and incubated at 37 °C for another 15 min. Subsequently, the cells were washed twice with PBS and harvested for flow cytometry analysis.

Isolation and culture of primary mouse hepatocytes

Primary cultured mouse hepatocytes were prepared from 12-week-old C57BL/6 mice and Wld^S^ mice by collagenase perfusion method as previously described [2]. Cells were plated with DMEM supplemented by 10% FBS, 100 units/ml penicillin and 0.1 mg/ml streptomycin.

Measurement of the isolated mitochondrial membrane potential

Mitochondria were isolated from livers of wild-type and Wld^S^ mice using the tissue mitochondria isolation kit (Beyotime Institute of Biotechnology) according to the manufacturer’s instructions. Mitochondrial membrane potential was measured with JC-1 staining as described with minor modifications [3]. Mitochondria were incubated in mitochondrial preserving buffer containing 5 µg/ml JC-1 dye for 20 min at 37 °C in the dark, and then washed twice and resuspended in mitochondrial preserving buffer containing the indicated concentrations of paraquat. After incubation for 15 min at room temperature, JC-1 red fluorescence was measured using a fluorescence plate reader (Flexstation II 384, Molecular Devices).

References

1. Ge X, Yu Q, Qi W, Shi X, Zhai Q (2008) Chronic insulin treatment causes insulin resistance in 3T3-L1 adipocytes through oxidative stress. Free Radic Res 42: 582-591.

2. Koo SH, Satoh H, Herzig S, Lee CH, Hedrick S, et al. (2004) PGC-1 promotes insulin resistance in liver through PPAR-alpha-dependent induction of TRB-3. Nat Med 10: 530-534.

3. Kluza J, Gallego MA, Loyens A, Beauvillain JC, Sousa-Faro JM, et al. (2006) Cancer cell mitochondria are direct proapoptotic targets for the marine antitumor drug lamellarin D. Cancer Res 66: 3177-3187.
